# Double Native Valve Infective Endocarditis due to *Capnocytophaga canimorsus*: First Reported Case Caused by a Lion Bite

**DOI:** 10.1155/2018/4821939

**Published:** 2018-02-08

**Authors:** Mazin Barry

**Affiliations:** Division of Infectious Diseases, College of Medicine, King Saud University, Riyadh, Saudi Arabia

## Abstract

*Capnocytophaga canimorsus* is a Gram-negative bacilli that is part of the normal oral flora of dogs and some cats; it is well known to cause septicemia and endocarditis after their bite. This is the first reported case of infective endocarditis affecting both native mitral and aortic valves after a lion bite in a patient with heavy ethanol consumption, who ultimately died after valve replacements.

## 1. Case History

**Figure 1:**
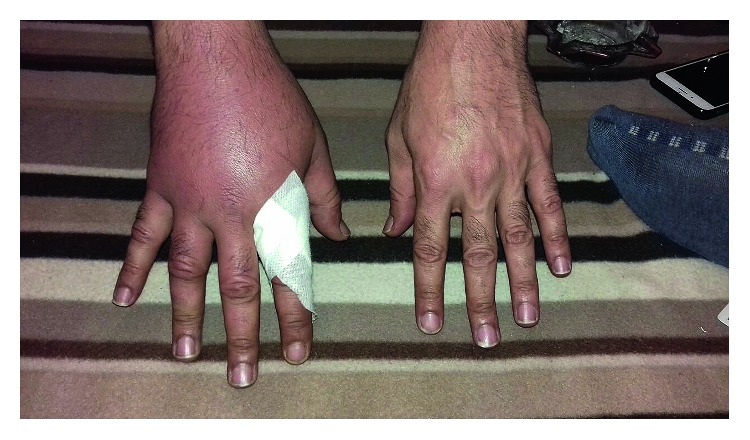
Swollen right hand with redness. This led to a diagnosis of cellulitis.

A 43-year-old man presented to our institution's emergency room in 2017 with fever for three weeks, which started one week after being bitten by a lion. A month ago while caring for this lion, he was bitten on the right hand at the web between the index and thumb. It was an unprovoked penetrating bite; he had to pull out his hand to set it free. He self-managed the injury. Next day his whole right hand had become red and swollen, and he visited an outpatient clinic where he received wound care and antitetanus injection; he was also diagnosed with cellulitis (Figure 1) and prescribed cefuroxime 500 mg PO q12 h for 10 days with complete resolution of the swelling, However, three days after stopping cefuroxime, he developed fever as high as 39°C, accompanied with chills, rigors, and sweating, which was continuous, lasting over three weeks. A few days before presentation, he noticed the pulp of his left fifth finger become red, swollen, and tender. This resolved spontaneously the next day.

He had no known past medical problems, no known liver disease, or splenic dysfunction. He worked as an animal caregiver for an exotic animal collector for the past three years. He was bitten by a 15-month-old African lioness, which was born into captivity in Saudi Arabia. The lioness was healthy and did not display abnormal behavior before nor after the bite. He had no contact with other animals including dogs or cats. He had consumed two to three alcoholic beverages per day for most of his adult life.

On physical examination, his temperature was 38.2°C, blood pressure (BP) 110/50 mmHg, pulse 110 beats/min, respiratory rate 16 breaths/min, and O_2_ saturation 97% on room air.

His right hand was completely normal with a healed bite mark but had multiple Janeway lesions on both palms and soles. Cardiac examination revealed a soft S1, normal S2, an early diastolic blowing murmur at the left upper sternal border, and an ejection systolic murmur. A pansystolic murmur was also audible at the apex. Abdominal examination did not show hepatosplenomegaly.

Laboratory tests showed white blood cells (WBCs) 19 × 10^9^/L (normal 4.0–11.0 × 10^9^/L), neutrophils 14.3 × 10^9^/L (normal 2.0–7.5 × 10^9^/L), hemoglobin 123 g/L (normal 130–180 g/L), hematocrit 39% (normal 42.0–52.0%), platelet 285 × 10^9^/L (normal 140–450 × 10^9^/L), erythrocyte sedimentation rate (ESR) 84 mm/h (normal 0–17 mm/h), C-reactive protein (CRP) 266 mg/L (normal <10 mg/L), international normalized ratio 1.2 seconds (normal 0.8–1.3 seconds), and creatinine 192 *µ*mol/L (normal 53–115 *µ*mol/L). Urinalysis showed +3 blood, 32/HPF red blood cells (RBCs), +1 protein, and urobilinogen negative. Electrocardiography revealed sinus tachycardia. Chest and right hand radiographs were both normal.

A diagnosis of infective endocarditis was made, and after blood cultures were drawn, he was started on intravenous ceftriaxone (2 g q24 h), gentamicin (70 mg q12 h), and vancomycin (1 g q24 h). A transesophageal echocardiography (TEE) showed that the aortic valve had a large 1.2 × 1 cm mobile mass attached to the left coronary cusp, which was perforated and destroyed, with severe aortic regurgitation. The mitral valve had a small 0.5 × 0.5 cm mass attached to the tip of the anterior leaflet, with moderately severe mitral regurgitation.

Clinically, on day three of admission, his fever resolved. The laboratory parameters had also improved: WBC 11.9 × 10^9^/L, ESR 63 mm/h, creatinine 137 *µ*mol/L, and urine RBC 3/HPF.

On day four of admission, all six bottles from all three sets taken on the day of admission grew Gram-negative bacilli. Later, it grew only on the chocolate agar, but not on MacConkey agar. The growth was oxidase-negative, catalase-negative, and arginine dihydrolase-positive. Definitive species identification was made by 16S ribosomal RNA sequencing. Universal 16S rRNA bacterial primers 27FYM [1] and 519R were used to amplify the V1–V3 region of the 16S rRNA gene as described previously [2] using 50 ng of genomic DNA. The PCR products were purified using QIAquick PCR Purification Kit (Qiagen) and sequenced using both forward and reverse primers. Sequencing was performed using BigDye™ Terminator v3.1 Cycle Sequencing Kits and 3730xl DNA Analyzer (Life Technologies, USA) according to the manufacturers' instructions. The sequence was subjected to BLAST analysis using the NCBI database, and the isolate was identified as *Capnocytophaga canimorsus*.

Direct susceptibility testing (using other non-Enterobacteriaceae minimum inhibitory concentrations (MICs)) showed cefotaxime MIC 0.023 mg/L, and for gentamicin, MIC was 96 mg/L. Vancomycin was immediately stopped after the initial Gram stain result, and gentamicin was stopped on day six of admission after the latter results; the patient was continued on ceftriaxone. Two sets of repeated blood cultures on day two of admission did not grow any organism.

**Figure 2:**
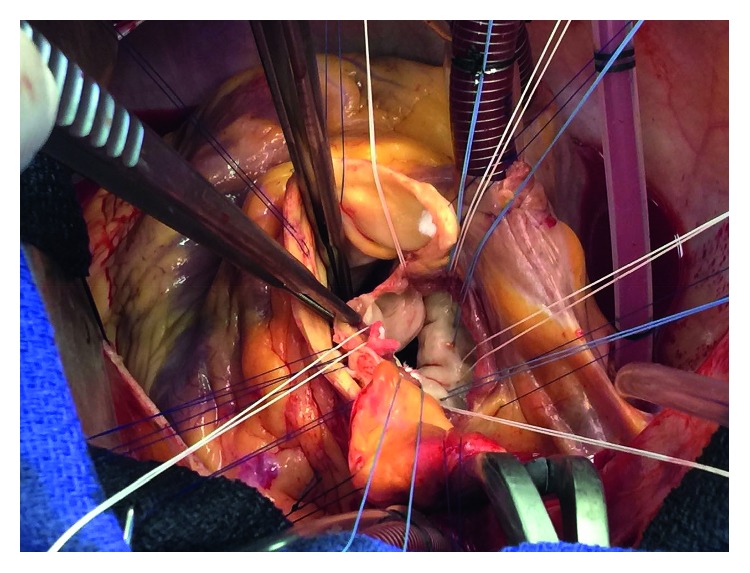
Cardiac surgery showing a perforated aortic valve.

On day seven of admission, he underwent mechanical aortic valve replacement, mitral valve repair with annuloplasty, reconstruction of the aortic-mitral curtain, debridement and excision abscess space in the aortic root, and coronary artery bypass grafting. It was noted that the left coronary cusp was perforated and destroyed by the infection with multiple vegetation threads (Figure 2). The next day, he died from direct complications of the surgery.

Valve tissue Gram staining did not show any organisms. No organisms grew in either solid or liquid mediums after three weeks of incubation even after blind subculture. Histopathology only showed fibrin, acute inflammatory exudate, and fragments of necrotic tissue which may explain the lack of organisms on Gram stain.

## 2. Discussion

This case highlights the importance of obtaining an infectious diseases consultation in cases with fever after animal bites, especially with exotic and unusual animals, in this case being a lion. It took more than 72 hours for C*apnocytophaga canimorsus* to grow in blood culture. It is a slow-growing but highly virulent organism, causing large vegetation in the aortic valve with perforation and abscess extending to the mitral valve. This patient was not known to have any heart disease prior to this event and was not known to have splenic dysfunction or chronic liver disease; however, he consumed alcoholic beverages in the form of *Araq*, which is a locally brewed Levantine spirit with high alcohol content (63% alc. vol./100 proof). This, in particular, made him at risk of developing complications, with respect to this bacterial species.

Zoonotic species of *Capnocytophaga* include *C. canimorsus* and C. *cynodegmi,* which are both normal inhabitants of the oral cavity of dogs and cats [3]. *C. canimorsus,* especially, may be on the rise because of more pet ownership, more animal contact, and improved laboratory methods for detecting infections [4]. It has a wide variety of manifestations including septicemia in patients with ethanol abuse (69%), splenectomy (33%), and immunosuppression (5%). Despite this, up to 40% of septicemia can occur in patients with no predisposing conditions [5]. Other less frequent infections include infective endocarditis. Sandoe reviewed 12 known cases in the literature over a period of twenty-five years, and twelve cases were published, four affecting the aortic valve, two affecting mitral valve, and one affecting both mitral and tricuspid valves [6]. Similarly Hayani et al. reported a case of double aortic and tricuspid valve endocarditis due to *Capnocytophaga canimorsus* after a dog bite [7] Contact with dogs or cats is the norm for cases with such infection, and no previous case has been linked to lions.

Suzuki et al. used polymerase chain reaction (PCR) to establish the prevalence of *Capnocytophaga* spp. in sheltered animals and found that 240 of 325 (74%) dogs and 66 of 115 (57%) cats tested positive for *C. canimorsus*, and *C. cynodegmi* was detected in 279 of 325 (86%) dogs and 97 of 115 (84%) cats. Both species were simultaneously detected in 219 of 325 (67%) of dogs and 64 of 115 (56%) of cats [8]. No similar studies have been done on lions.

If clinicians suspect *C. canimorsus* infection, the microbiology laboratory should be alerted for blood culturing for long periods of up to 10 days to enhance its chance of recovery. *C. canimorsus* is a fastidious and slow-growing organism that needs blood-enriched media and generally requires an environment with increased CO_2_ (5% to 10%). They grow on blood agar and often on chocolate agar, but not on MacConkey or heart infusion agars [9]. A number of methods are used to identify isolates to species level, including conventional biochemical substances, protein profiles, multilocus enzyme electrophoresis, serotyping of immunoglobulin A1 proteases, DNA probes, 16S rRNA PCR restriction fragment length polymorphism analysis, and 16S rRNA gene sequencing [10].

*C. canimorsus* is generally susceptible to many antibiotics. Some strains produce *β*-lactamase; thus, a penicillin–*β*-lactamase inhibitor combination or a third-generation cephalosporin is the first drug of choice for parenteral therapy. There is no standardized method of susceptibility testing for *Capnocytophaga* spp. The different methods used by different researchers may explain the varying susceptibility results described in the literature for the same antimicrobial agents [11].

In conclusion, this case is the first reported case of infective endocarditis caused by a lion bite, in general and specifically, due to *C. canimorsus*, which caused severe, double left-sided native valve disease requiring urgent cardiac surgery. Clinicians should be aware of such risk from wild animals kept in captivity and should be able to inform high-risk groups, such as those with ethanol abuse, of the possibility of such disease. They should also be able to recognize and treat this condition early to prevent morbidity and mortality.
